# Point-of-Care Diagnostics for Improving Maternal Health in South Africa

**DOI:** 10.3390/diagnostics6030031

**Published:** 2016-08-31

**Authors:** Tivani P. Mashamba-Thompson, Benn Sartorius, Paul K. Drain

**Affiliations:** 1 Discipline of Public Health, School of Nursing and Public Health, University of KwaZulu-Natal, Durban 4001, South Africa; Sartorius@ukzn.ac.za; 2 International Clinical Research Center, Department of Global Health, University of Washington, Seattle, WA 98195, USA; pkdrain@uw.edu; 3 Division of Infectious Diseases, Department of Medicine, University of Washington, Seattle, WA 98195, USA; 4 Department of Epidemiology, University of Washington, Seattle, WA 98195, USA; 5 Department of Surgery, Massachusetts General Hospital, Boston, MA 02114, USA

**Keywords:** HIV, maternal health, point-of-care diagnostics, resource-limited settings

## Abstract

Improving maternal health is a global priority, particularly in high HIV-endemic, resource-limited settings. Failure to use health care facilities due to poor access is one of the main causes of maternal deaths in South Africa. “Point-of-care” (POC) diagnostics are an innovative healthcare approach to improve healthcare access and health outcomes in remote and resource-limited settings. In this review, POC testing is defined as a diagnostic test that is carried out near patients and leads to rapid clinical decisions. We review the current and emerging POC diagnostics for maternal health, with a specific focus on the World Health Organization (WHO) quality-ASSURED (Affordability, Sensitivity, Specificity, User friendly, Rapid and robust, Equipment free and Delivered) criteria for an ideal point-of-care test in resource-limited settings. The performance of POC diagnostics, barriers and challenges related to implementing POC diagnostics for maternal health in rural and resource-limited settings are reviewed. Innovative strategies for overcoming these barriers are recommended to achieve substantial progress on improving maternal health outcomes in these settings.

## 1. Introduction

Improving maternal health and preventing mortality in resource-limited settings is a global health priority. Resource limited settings are defined as settings with inadequate human resource, poor economic gains, and incomplete infrastructure. The World Health Organization (WHO) defines maternal health as the health of women during pregnancy, childbirth and the postpartum period [1]. A report on maternal deaths in South Africa noted that the majority (67%) of preventable maternal deaths were caused by non-pregnancy related infections, obstetric hemorrhage and complications of hypertension in pregnancy [2]. HIV testing forms a major part of primary health care and routine antenatal care in South Africa [3], with the estimated national HIV prevalence amongst pregnant women having increased by 0.2% from 2010 to 30% in 2013 [2]. The United Nations’ Sustainable Development Goal 3 (SDG 3) calls for more efforts towards fighting against new HIV/AIDS infections and maternal deaths [4]. Moreover, there is a recognized need to work towards the new The Joint United Nations Programme on HIV/AIDS (UNAIDS treatment targets of “90 × 90 × 90 by 2020” which include: 90% of all people living with HIV knowing their status; 90% of all people diagnosed with HIV on sustained antiretroviral therapy, and 90% of all people receiving antiretroviral therapy having viral suppression [5]. Significant gaps are still to be overcome in order to reach each of the above targets, particularly in high HIV prevalence and resource-limited settings. Challenges of the health care system, failure to use health care facilities, inadequate services and substandard care related to the knowledge and skill of the health care providers have also been associated with the high maternal deaths in South Africa [6]. Poor access to healthcare and to health inequity are also major problems in rural and remote communities [6,7].

The UNAIDS and their partners recently launched the diagnostics access initiative [8]. This initiative emphasises the need to develop innovative strategies that expand access to affordable diagnostic assays followed by immediate access to effective prevention, treatment, and care programs [8]. As a result, a new generation of diagnostic tests are being developed for use at the point-of-care (POC) that will not require a laboratory [9,10,11,12]. POC diagnostics have previously been defined based on geographic, functional, technological or operational context [13]. In the current review, the definition of POC test from Drain et al. and Pai et al. is adopted, where it is defined based on how and where the test or device is used [14,15]. POC testing is therefore defined as a diagnostic test that is carried out near patients and leads to rapid clinical decisions [14,15,16], with prompt access to these tests being encouraged in resource-limited settings [14]. WHO recommends that pregnant women be tested for HIV during their first antenatal clinic visit, and that all women who test positive are immediately initiated into a prevention of mother to child transmission (PMTCT) of HIV program [17,18]. Despite the substantial delivery of HIV prevention and treatment programs for antenatal patients in South Africa, rural communities in some areas of the country continue to experience an increase in HIV prevalence amongst pregnant women [19].

Essential interventions for pregnant women in Africa include identifying and managing obstetric complications, such as preeclampsia, tetanus toxoid immunization, intermittent preventive treatment for malaria during pregnancy (IPTp), as well as infections, such as HIV, syphilis and other sexually transmitted infections (STIs) [20]. A number of diagnostic tests have been incorporated as part of the antenatal care for low- and intermediate-risk women to help improve their health outcomes. The impact of POC diagnostics has been demonstrated on maternal patients in a variety of desired outcomes including: Enabling rapid diagnosis and access to anti-retroviral treatment (ART) [21]; prevention of mother to child transmission (PMTCT) of HIV [22]; increasing ART initiation rate [23]; and reducing the numbers of clinic visits [24]. Our objective was to evaluate a selection of essential antenatal test for their accessibility, availability and usage at POC in resource-limited settings. To achieve this, we compared currently recommended diagnostics testing for pregnant women in South Africa with that of the United States of America, which is reported to have a maternal mortality ratio (MMR) of <10% of South Africa [2,25]. The current and immerging POC diagnostics for maternal health are reviewed, with a specific focus on test availability, acceptability and utility in terms of the WHO quality-ASSURED (Affordability, Sensitivity, Specificity, User friendly, Rapid and robust, Equipment free and Delivered) criteria of an ideal point-of-care test in resource-limited settings [14] (Table 1). The performance of POC diagnostics, barriers and challenges related POC diagnostics in rural and resource-limited settings is discussed, and strategies are recommend to ensure the sustainability of POC diagnostic services to improve maternal health outcomes.

## 2. Recommended of Diagnostics for Antenatal Patients at in South Africa

The South African guidelines for maternity care recommend a list of routine and non-routine diagnostic tests for pregnant women in healthcare settings [28]. These guidelines were introduced as part of a strategy to improve the quality of care for maternal patients, and to lower the maternal and perinatal morbidity and mortality rates in South Africa [28]. The South African guidelines are compared with those of the United States of America (USA) [29]. Table 2 indicates the differences in the total number and types of tests that are offered routinely and non-routinely to pregnant women, with fewer being offered in USA (*n* = 17) than in the South Africa (*n* = 18).

All seven routine test recommended by the South African guidelines are available at POC, of which; two are for detecting communicable diseases (HIV and syphilis rapid test). Of the 12 tests routinely offered to pregnant women in the USA, nine are for communicable diseases, this being considerably more than the number offered in South Africa. South Africa offers pregnant women 11 non-routine tests, of which four are for communicable diseases. The USA offers four non-routine tests of which two are for communicable diseases. Bearing in mid the reported double disease burden of communicable and non-communicable disease in South Africa [30] and poor healthcare access for pregnant women in rural and resource-limited settings [6,7], there is an urgent need to increase the availability and use of diagnostic tests for both communicable disease and non-communicable diseases.

POC tests can be used in different settings and areas of clinical medicine, including maternal health care to facilitate early diagnosis and management of complex disease conditions [9,11,31]. However, in order to achieve the greatest impact, POC diagnostic programs must be carefully planned and implemented within a specific context [32]. WHO therefore developed guidelines to ensure that the POC diagnostics address the needs of the user in a clinically and cost effective manner, and to avoid the use of possibly expensive devices that fail to deliver the required outcomes [33]. These guidelines are intended for developing POC testing devices to detect sexually transmitted infections (STI), a major health problem in the developing world, and in the developed world, for diseases, such as Chlamydia and HIV [33]. Table 1 shows the criteria of ideal characteristics for a POC test in resource-limited settings [14,26,27].

## 3. Current and Emerging POC Diagnostics for Maternal Health

POC diagnostics are currently available in resource-limited settings for routine and non-routine maternal health related tests, and are currently applied in a variety of settings, from homes to hospitals [34]. The tests can also be used in many areas of clinical medicine, including maternal healthcare, to facilitate early diagnosis and management of complex disease conditions [9,11,31]. Here we discuss current and immerging POC test with potential to improved healthcare access and maternal outcomes of HIV infected women in settings that lacks laboratory infrastructure.

### 3.1. Pregnancy Test

In South Africa, women and girl who are suspected of being pregnant and HIV infected are advised to have a pregnancy test, which are free at primary healthcare (PHC) clinics and other health facilities. Home-based pregnancy testing kits are also accessible from commercial pharmaceutical outlets, but may not be accessible or affordable for those in rural setting. Early detection of pregnancy in HIV infected women can allow prompt enrolment in care within the PMTCT program [35]. The urine pregnancy test is also important for family planning [36]. The WHO recommends that health workers confirm that a woman is not pregnant before offering her hormonal contraceptives [37]. This need to verify the pregnancy status presents a barrier to increasing contraceptive use due to lack a reliable testing methods in some rural and resource-limited settings [38], highlighting need for improved access for pregnancy tests for affected women, particularly in HIV endemic regions.

An early (1998) meta-analysis aimed at assessing the performance of home pregnancy test (HPT) kits, where test results of actual patients were compared to those produced by diagnostic trained volunteers revealed a relative low effectiveness score of HPT kits when used by actual patients [39]. The test effectiveness score was 2.75 (95% confidence interval (CI): 2–3) for studies which involved trained volunteers but deteriorated to 0.82 (CI: 0–1) for studies which involved actual patients [39]. These results demonstrated that HPT diagnostic performance is greatly affected by characteristics of the users.

### 3.2. Blood Glucose Test

**Figure 1:**
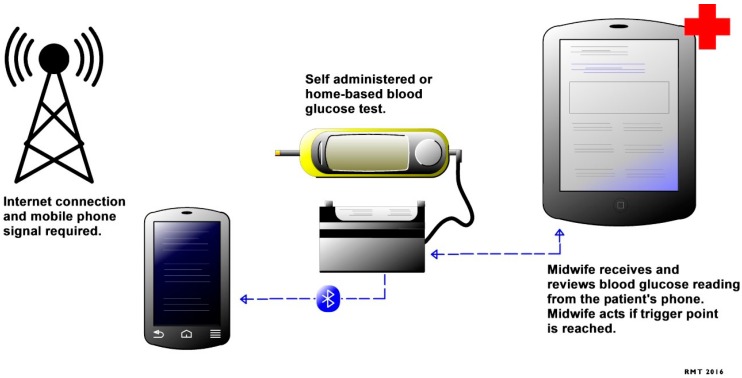
Mobile health system for management of gestational diabetes mellitus adapted from Mackillop et al., 2014 [45].

WHO recommends blood glucose testing as an essential diagnostic tool during pregnancy [40]. Blood glucose tests are used to detect gestational diabetes mellitus, which is characterized by elevated glucose intolerance during pregnancy. Raised glucose levels have been associated with poor maternal and child outcomes [41,42], as well as obesity, which is one of the main causes of mortality globally [43]. Regular clinical assessment of patients with gestational diabetes mellitus by healthcare professionals is required, due to the rapidly changing physiology of pregnancy and its unpredictable course. Blood glucose tests are currently offered to patients as a facility- and home-based test. Mobile health technology aimed at improving healthcare access for women with gestational diabetes mellitus and to enable home-based blood glucose monitoring is currently under development, with the results being transmitted in real time to a healthcare professional (Figure 1) [44,45]. This novel technology is aimed at lowering the cost to the patient and healthcare provider by reducing the number of clinic visits for women with gestational diabetes and those at high risk of developing gestational diabetes [44,45]. This will offer a great healthcare opportunity for healthcare providers and patients in resource-limited settings, due to the reported staffing challenges and poor healthcare access for pregnant women.

### 3.3. HIV Testing

Infectious diseases, including HIV, constitute 11% of global maternal deaths [46]. In South Africa, approximately 10,000 new HIV infections per year were reported between 2012 and 2016 [47]. HIV testing is a gateway to HIV/AIDS prevention, care, and treatment as well as support interventions. The HIV rapid diagnostic test is widely available and offered as an essential test for pregnant women during their first antenatal clinic visit (Table 1). Rapid HIV testing is one of the successful strategies for improving healthcare access and maternal health outcomes [22,48,49,50,51,52]. Male HIV testing has also been proven to help improve maternal and child outcomes for HIV infected patients [53]. However, HIV testing uptake by partners of pregnant women is low, leading to poor maternal and child outcomes [54]. Strategies to increase adoption of HIV testing by high-risk populations, such as pregnant women and their partners, are encouraged. One such strategy is HIV oral self-testing, which is currently available in the USA, and has been shown to offer convenience and privacy, with the potential to increase the number of people who test regularly [55]. An investigation to determine the feasibility of implementing acceptable POC HIV self-tests in resource-limited settings has revealed common errors in performing the tests and poor result interpretation [56]. Therefore, adaptation of future HIV self-test kits instructions needed for low-literacy participants has been recommended to ensure utility of this test for resource-limited settings [56].

A comparative study to determine the diagnostic accuracy of a rapid HIV-antibody-based point-of-care test (Oraquick advance rapid HIV-1/2, OraSure Technologies Inc., Bethlehem, PA, USA) for oral and blood-based specimens in adults was conducted [57]. The results of the study showed a lower (2%) pooled sensitivity in oral specimens, 98% (CI: 96%–99%) than in blood-based specimens, 100% (CI: 97%–100%) [57]. However, the sensitivity was similar, oral 100%, (CI: 99%–100%); blood 100%, (CI: 100%–100%), in both specimen types [57]. The Oraquick had also showed a high positive predictive value (PPV) in high HIV prevalence settings in oral specimens, the slightly lower sensitivity and PPV in low-prevalence settings in oral specimens [57]. When evaluated in a healthcare setting, rapid HIV tests were less sensitive on oral fluid than on finger-stick whole blood and less sensitive on finger-stick whole blood than on serum [58]. This calls for a careful review of the use of oral specimens in HIV testing prior to adoption, particularly in high HIV prevalence regions.

### 3.4. CD4 Cell Count Test for HIV Monitoring

The WHO recommended that women who are newly diagnosed as HIV-positive during pregnancy, and those HIV-infected women who become pregnant and are not on ART, be offered a CD4 count test as part of WHO staging and determination of the appropriate ART regime [59]. The CD4 count test was previously an essential test for HIV-infected women as part of the PMTCT HIV program in South Africa. However, the South African Department of Health recently adopted the WHO B+ HIV prevention approach for all pregnant women who test positive [57,60]. The WHO B+ approach is a simplified program that enables integration of PMTCT and ART at the primary care level [17]. This approach entitles every pregnant and breastfeeding woman to lifelong antiretroviral therapy (ART) regardless of their CD4 count or clinical staging [57,60].

The use of laboratory based CD4 count tests has led to diagnostic delays, loss to follow-up, and missed opportunities for PMTCT [61]. A study reports high rate of transmitted antiretroviral (ARV) drug resistance amongst HIV infected women in rural South Africa [62], which can abrogate drug efficacy [63]. Despite the change of policy in HIV management for HIV infected women [57,60] improved accessibility is required for CD4 count testing at POC to enable rapid determination of ARV efficacy and treatment regime change to ensure PMTCT for maternal patients. The impact of CD4 count on POC diagnostics has been demonstrated in reduced pre-treatment loss to follow-up and increased ART initiation rates for HIV infected patients in resource-limited settings [23,64,65]. Task-shifting and integrating POC CD4 cell count testing component in HIV positive mother and child healthcare facilities to allow effective co-management of pregnancy and HIV in the same site has been proposed [66].

The results of the consortium which conducted a pooled multi-data technical performance analysis of the Pima CD4 on venous and capillary samples has shown acceptable performance of the devise with a sensitivity of 93%, CI: 91%–95%) at 350 cells/μL and 96% (CI :95%–97%) at 500 cells/μL [67]. Moreover, no significant difference between venous and capillary testing [67]. However, the sensitivity decreased to 86% (CI 82%–89%) at 100 cells/μL (for Cryptococcal antigen (CrAg) screening), with a significant difference between venous 88% (CI: 85%–91%) and capillary 79% (CI: 73%–84%) testing [65]. The results also show no consistency in Pima CD4 misclassification between the meta-analysis data and a population subset of HIV+ and ART naïve individuals as well as misclassification among operator cadres [65]. Careful planning and judicious system-building for implementing CD4 count testing have been suggested for resource-limited settings to ensure reliability and accuracy of the results [68].

### 3.5. HIV Viral Load Test

**Figure 2:**
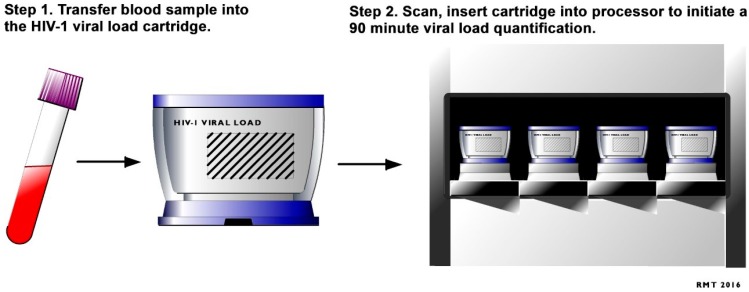
HIV-1 Viral Load quantitative point-of-care testing process adapted from Cepheid Solutions [71].

HIV viral load monitoring is recommended by WHO as the principal test for efficacy of ART treatment, and to determine if HIV positive women should have a C-section rather than a vaginal delivery [69]. An elevated HIV viral load (>1000 IU/mL) can put infants at risk of HIV transmission and other T-cell related abnormalities during the birth process [70]. HIV viral load monitoring is currently provided by laboratories and requires plasma samples in most resource-limited settings. Improving access to viral load testing to antenatal clinic though the introduction of POC HIV viral load tests, such as the recently approved Xpert^®^ HIV-1 VL [71], can improve early detection of drug resistance in rural antenatal clinic that have poor access to laboratory infrastructure. POC viral load testing can also enable timely diagnosis of acute HIV infection at clinics where patients at high risk can then receive care [71]. Use of POC viral load in these settings has the potential to enhance the effectiveness of PTMCT programs in order to meet the United Nations treatment target to achieve viral suppression in 90% of people on ART by 2020 and end the AIDS pandemic [4]. Patient-centered benefits of the emerging semi-quantitative POC viral load assays, which detect HIV RNA using a lateral flow dipstick with a novel isothermal amplification technique, has been demonstrated [46]. The need for POC HIV viral load diagnostics has been highlighted in SA [46], with simple testing being key to enabling the implementation of POC HIV viral load technologies to remote or resource limited settings [71]. A new fully automated real-time molecular-based HIV-1 VL POC test, Cepheid GeneXpert^®^ (Xpert, Sunnyvale, CA, United States) HIV-1 viral load (VL) assay received European CE-IVD regulatory approval in December 2014 [71]. The Xpert HIV-1 VL POC test has recently been validated for use in clinical settings in South Africa [72]. This is an easy to use test which, enables RNA extraction, purification, reverse transcription and cDNA real time quantitation in one fully integrated cartridge and produce results in less than two hours (Figure 2) [71]. The results of the validation showed that the Xpert^®^ HIV-1 VL has good correlation with an established laboratory based viral load assay. This demonstrates that the Xpert^®^ HIV-1 VL POC test could be a reliable tool for clinic-based viral load monitoring in clinical settings in South Africa [72]. These results are important and a first step to implementation of HIV VL POC testing in South Africa.

### 3.6. Syphilis Testing

The WHO recommends syphilis screening for pregnant women as part of the delivery of maternal and neonatal care in health facilities [73], and is one of the essential antenatal tests for improving maternal and child morbidity and mortality [74]. Despite this, it has been demonstrated that up to one third of the women attending antenatal care (ANC) clinics globally are not tested for syphilis [75]. Syphilis POC diagnostics are currently available, have shown good reliability and can be performed in any clinical setting [76]. Research has shown the effectiveness of POC immunochromatographic test to simultaneously detect both non-treponemal and treponemal antibodies in the sera of patients with syphilis that acts as both a screening and a confirmatory test for use in resource-limited settings [77]. In addition, studies have shown the effectiveness of syphilis POC testing on improving maternal and child outcomes [24,50,78].

### 3.7. Chlamydia, Gonorrhoea

*Chlamydia trachomatis (CT)* and *Neisseria gonorrhoeae (NG)* are well established agents of sexually transmitted infections (STIs), which have been associated with adverse maternal outcomes, particular for HIV infected women and their offspring [79,80]. Immerging POC tests which offer a molecular based approach to diagnosing these STIs, Cepheid GeneXpert^®^ (Xpert) CT/NG assay was US FDA-cleared in December 2012 [81]. This test has been cleared for use in female endocervical swabs, patient-collected vaginal swabs and for female and male urine specimens from both symptomatic and asymptomatic patients [81]. The accuracy of this test has been evaluated and the results demonstrated an acceptable sensitivity for chlamydia using endocervical, vaginal, and urine samples, 97.4%, 98.7%, and 97.6%, respectively and ≥99.4% specificity [82]. When using female and male urine a sensitivity was estimated at 97.5% and specificity at ≥99.4% [82]. Results for the gonorrhea test demonstrated sensitivities for endocervical, vaginal, and urine samples of 100.0%, 100.0%, and 95.6%, respectively, in females with specificity at ≥99.8% [82]. However, in male urine specimens, sensitivity was estimated at 98.0%, and specificity at ≥99.8% [82]. The clinical implications of introducing the Xpert CT/NG assay in settings with poor access to laboratory infrastructure have been demonstrated [83]. The identified benefits of introducing this POC test include the following: more timely and targeted prescribing, to reduce *Neisseria gonorrhoeae* drug resistance [84]; freeing up staff time usually spent on follow-up of cases who haven’t returned for treatment; greater opportunities to offer a full STI screen for people with a positive test; more timely and targeted contact tracing; and more timely information to guide pelvic inflammatory disease diagnosis and management [83]. Despite the potential impact that this POC testing would have on different stages of the *Chlamydia trachomatis* and *Neisseria gonorrhoeae* management pathways, careful evaluation of testing and disease management cascade, is recommended [83].

### 3.8. Hepatitis B Virus

Hepatitis B virus (HBV) infection is the most serious type of viral hepatitis, which leads to a potentially life-threatening liver infection, chronic liver disease and liver cancer [85]. Perinatal hepatitis B transmission is strongly associated with Hepatitis B “e” antigen (HBeAg) positivity of childbearing women [28]. HBV and HIV co-infection have been associated with an increased risk for liver-related deaths [86]. HBV POC tests, such as Determine, Vikia, and Espline, are currently available in the market [87]. Validation of HBV POC tests (Determine, Vikia, and Espline) for the detection of HBsAg at a community level in a resource-limited setting has shown that these tests represent accurate, rapid, and inexpensive alternatives to serology testing for the screening of HBV infection [87]. Prompt access to these diagnostics in high HIV prevalence regions such as South Africa, is recommended.

## 4. Discussion

The introduction of POC diagnostics has revolutionised medical care, with numerous research findings having demonstrated the impact of POC diagnostics on improving maternal health outcomes [21,22,48,49,50,51,52]. It is evident that diagnostics form a fundamental part of antenatal health care services, in South Africa. POC diagnostics tests for detection of HIV infection, pregnancy, syphilis infection, rhesus D blood group, haemoglobin, urine and glucose are offered as part of the routine antenatal care in South Africa. However, despite the high national HIV prevalence [88], launch of the diagnostics access initiative by the Joint United Nations Programme on HIV/AIDS (UNAIDS) and partners [11] and the new UNAIDS treatment targets of “90 × 90 × 90 by 2020” [40], pregnant women in South Africa have limited access to essential tests for HIV management as part of their routine care (Table 2). A comparison of the number of routine and non-routine antenatal diagnostic tests in SA and USA has revealed low levels of communicable disease tests offered in South Africa. Efforts to implement HIV management using POC tests in South Africa has been demonstrated through a study aimed at determining the accuracy of Xpert^®^ HIV VL POC test [72].

As demonstrated in Table 2, *Chlamydia trachomatis* and Hepatitis B virus tests are offered as part of routine antenatal diagnostic test in the USA but this is not the case in South Africa. STIs, such as *Chlamydia trachomatis* and Hepatitis B virus infections, are associated with adverse maternal outcomes [79,80,85]. In addition, gestational diabetes mellitus has been closely associated with adverse maternal outcomes due to its effect on maternal insulin secretion and temporary metabolic stressors imposed by the placenta and foetus, both South Africa and the USA do not offer blood glucose as a routine diagnostic test. Bearing in mind the reported number of STI-related maternal deaths in South Africa [2] and the metabolic and mitochondrial effects of antiretroviral drug exposure during pregnancy and postpartum [89], we recommend the introduction of *Chlamydia trachomatis*, Hepatitis B virus and blood glucose POC tests as part of the routine diagnostic antenatal test in South Africa.

The use of POC tests that are suitable for home-based or self-testing have raised some concern with regards to performance and reliability of results [58]. Innovative programmes that combine eHealth and home-based testing have been shown to help improve access to disease testing and healthcare [90]. This approach can also help empower patients, particularly for stigmatized diseases such as HIV/AIDS [33]. There is a need to overcome these challenges by introduction of affordable, user friendly molecular-based tests to ensure delivery of quality-assured POC diagnostics services. These tests have potential to offer great benefits to the most vulnerable populations, such as rural and remote community with poor access to health facilities.

**Figure 3:**
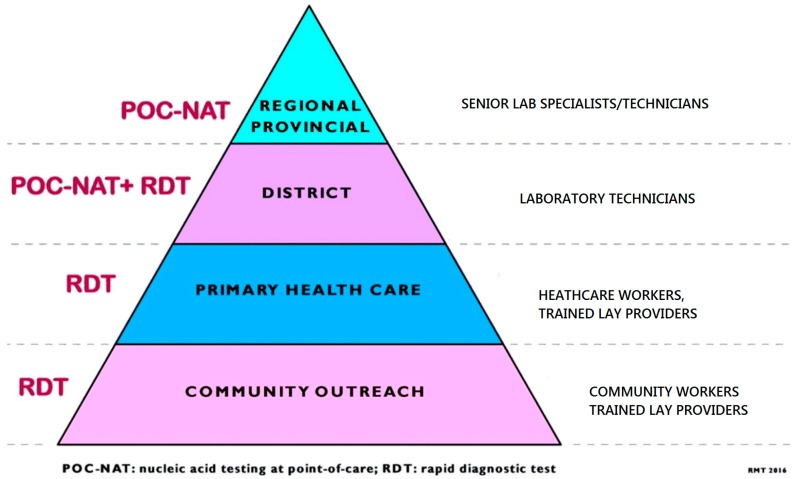
Pyramid of point-of-care diagnostic testing. Adapted from WHO pyramid of laboratory testing [91].

A significant increase in public and private sector investment in HIV/AIDS and TB POC diagnostics for resource-limited settings has been shown in recent years. However, the complexity of the assay that can be used in a given setting (Figure 3) is determined by the type of infrastructure and human resource required [18]. Some of the challenges relating to implementation of POC testing are economic, policy-related regulatory, laboratory capacity, infrastructure, quality control and quality assurance, work-flow balance, training, supply chain, infection risk, administrative/operational, technical/medical, awareness, health systems-related, and cultural/societal [15]. Due to challenges relating to implementation of POC diagnostics in resource-limited settings, the majority of patients in resource-limited settings do not have access to the essential and more accurate POC diagnostic tests at community and PHC level [91], which potentially undermines their impact of POC on patient outcomes. Bearing in mind the need for improving diagnostic testing for key populations such as HIV infected pregnant and breast feeding patients, for POC diagnostics to be useful, they must be accurate, simple and affordable for the population for which they are intended [92,93]. The recent adoption of the United Nations SDG 3 in South Africa has the potential to prompt the increased availability of POC diagnostics in rural and resource-limited settings for HIV [4]. As the use of POC diagnostics expands to resource-limited settings, appropriate strategies need to be adopted to address these barriers to ensure efficacy and sustainability of the technology.

Numerous emerging POC tests with potential to improve maternal health outcomes have been discussed in this review. For POC diagnostics to be useful, they must be accurate, simple and accessible for use by the population for which they are intended [92,93]. The usefulness of the test in a given setting can only be determined by conducting test evaluations [92], which incorporate performance and operational characteristics [92]. Action is needed to improve the performance, accessibility and usage of new POC tests for both communicable and non-communicable diseases to help improve maternal health outcomes in high disease prevalence such as South Africa. We recommend the following strategy to help improve the performance, accessibility and usage of POC diagnostic services in resource-limited settings with limited access to laboratory infrastructure: First, introduction of a new dedicated cadre of health workers for POC diagnostics services; implementation of an adaptable supply chain management system that includes agility sustainability; and a regular detailed evaluation of POC diagnostic services. Implementation of the new strategies should be conducted as a step-by-step process which is supported by development of appropriate local policies and tools. To ensure sustainability of the recommended approaches, key stakeholder collaboration and guidance under the leadership of the ministries of health, will be essential.

## 5. Conclusions

The advent of POC diagnostics provides a promising hope to improving maternal health outcomes, particularly to HIV epidemic regions. Overcoming challenges related to the accessibility, availability and utility of POC diagnostics for high-risk populations should be prioritized prior to adapting or adopting new POC tests. To ensure the successful implementation of new diagnostics to address the unmet needs of patients in resource-limited settings, evidence based interventions to overcome the above challenges are urgently needed.

## Figures and Tables

**Table 1:** The ASSURED (Affordability, Sensitivity, Specificity, User friendly, Rapid and robust, Equipment free and Delivered) criteria for ideal point-of-care (POC) diagnostics for use in resource-limited settings [14,26,27].

Criteria	Characteristic
Affordable	Purchasable price for settings comprised of population at risk of infection
Sensitive	Results contain minimal false negatives (99%)
Specific	Results contain minimal false positives (99%)
User-friendly	Required minimal steps to carry test
Rapid and robust	Short turnaround time and no need for refrigerated storage
Equipment free	No need for no complex equipment
Delivered	Made accessible to end users

**Table 2:** Tests recommended for pregnant women in South Africa (SA) [28] and the USA [29].

**Routine Tests**			
**South Africa**		**USA**	
***Test***	***Communicable (Yes/No)***	***Test***	***Communicable (Yes/No)***
Pregnancy test	No	Pregnancy test	No
Syphilis serology	Yes	Blood type and Rh factor	No
Rhesus (D) blood group	No	Anemia	No
Hemoglobin (Hb) level	No	Hepatitis B and C	Yes
HIV serology	Yes	Syphilis	Yes
Urine protein	No	HIV serology	Yes
Urine glucose	No	Chlamydia	Yes
		Chicken pox	Yes
		Rubella	Yes
		Gestational diabetes	Yes
		Toxoplasmosis	Yes
		Ultrasound scan	No
		Group B Streptococci	Yes
**TOTAL**	**2**		**9**
***Non-Routine Tests***			
**South Africa**		**USA**	
***Test***	***Communicable (Yes/No)***	***Test***	***Communicable (Yes/No)***
ABO blood group	No	Down’s syndrome screening	No
Down’s syndrome screening	No	Viral load	Yes
Rubella serology (German measles)	Yes	CD4 count	No
Blood glucose screening	No	TB	Yes
CD4 count	No		
TB	Yes		
Creatinine	No		
HIV Viral load	No		
Ultrasound scan	No		
Urine culture	Yes		
Cervical (Papanicolaou) smear	Yes		
**TOTAL**	**4**		**2**
